# The Association between Serum Adiponectin Levels and Endothelial Function in Non-Dialysis-Dependent Chronic Kidney Disease Patients

**DOI:** 10.3390/biomedicines11082174

**Published:** 2023-08-02

**Authors:** Ming-Chun Chen, Chung-Jen Lee, Yu-Li Lin, Chih-Hsien Wang, Bang-Gee Hsu

**Affiliations:** 1 Department of Pediatrics, Hualien Tzu Chi Hospital, Buddhist Tzu Chi Medical Foundation, Hualien 97004, Taiwan; loveroflois1980@gmail.com; 2 School of Medicine, Tzu Chi University, Hualien 97004, Taiwan; nomo8931126@gmail.com (Y.-L.L.); wangch33@gmail.com (C.-H.W.); 3 Department of Nursing, Tzu Chi University of Science and Technology, Hualien 97005, Taiwan; guggilee@msn.com; 4 Division of Nephrology, Hualien Tzu Chi Hospital, Buddhist Tzu Chi Medical Foundation, Hualien 97004, Taiwan

**Keywords:** adiponectin, chronic kidney disease, digital thermal monitoring test, endothelial function, vascular reactivity index, blood-vessel myography

## Abstract

Adiponectin is the richest human circulating adipokine with anti-inflammatory, antioxidant, and insulin-sensitizing effects. We evaluated the association between serum adiponectin levels and endothelial function in chronic kidney disease (CKD) patients, obtaining fasting blood samples from 130 non-dialysis CKD subjects. We measured the endothelial function—represented by the vascular reactivity index (VRI)—via non-invasive digital thermal monitoring, and serum adiponectin concentrations by enzyme immunoassay kits. A total of 22 (16.9%), 39 (30.0%), and 69 (53.1%) patients had poor (VRI < 1.0), intermediate (1.0 ≤ VRI < 2.0), and good (VRI ≥ 2.0) vascular reactivity. Elevated serum blood urea nitrogen (BUN) level was negatively correlated with VRI values, but serum adiponectin and estimated glomerular filtration rate were positively associated with VRI values by univariate linear regression analysis. After applying multivariate stepwise linear regression analysis adjustment, the significantly positive association of adiponectin (*p* < 0.001), and the significantly negative association of log-BUN (*p* = 0.021) with VRI values in CKD subjects remained. In an animal study using in vitro blood-vessel myography, treatment with adiponectin enhancing acetylcholine-mediated vasorelaxation in 5/6 nephrectomy CKD mice. Our study results indicated that adiponectin concentration was positively associated with VRI values and modulated endothelial function in non-dialysis CKD patients.

## 1. Introduction

Chronic kidney disease (CKD)—one of the most critical risk factors for markedly increased cardiovascular disease (CVD) known as CKD progression—is currently a major global health problem [1]. In CKD patients, endothelial dysfunction (ED), an early step of atherosclerosis is an essential nontraditional cardiovascular (CV) risk factor contributing to CV events [2]. The ED assessment could usefully predict CV disease not only in the general population but also in high-risk subjects, including CKD patients [3].

All CKD patients, from pre-dialysis to maintenance peritoneal dialysis and hemodialysis (HD), are reported in the body of literature to exhibit ED [4,5,6], which—even in the early stage of CKD—contributes to arterial stiffness and renal interstitial fibrosis independent of hypertension [6,7]. Uremic toxin accumulation has been demonstrated to modulate the process of ED [8]. The previous literature revealed an inverse association between indoxyl sulfate levels and ED in the late CKD population evaluated by vascular reactivity index (VRI) values [9]. A recent study also showed that the endothelial glycocalyx—a layer overlying the endothelial lining of all vessels, characterized as a novel early endothelial damage biomarker—is markedly increased in CKD patients with higher uremic toxin levels [10].

Adiponectin, secreted mainly from white adipose tissue, and the richest human serum adipokine with insulin-sensitizing, anti-inflammatory, anti-atherogenic, and antioxidant effects, plays a central role in CV disease protection [11]. Many animal and human studies support the association between hypoadiponectinemia and ED [12,13]. Impaired nitric oxide (NO) production and endothelium-dependent vasodilatation were reported in adiponectin-deficient mice [12], and diabetic patients with hypoadiponectinemia had inadequate vasodilatory responses [13]. Adiponectin presented its anti-inflammatory effects via inhibiting nuclear factor-kappa B activation in the vascular endothelium [11]. Adiponectin can—by activating with intracellular protein adaptor protein phosphotyrosine interacting with pleckstrin homology domain and leucine zipper 1 (APPL1)—enhance fatty acid oxidation and augment the vasodilatory effect of endothelial nitric oxide synthase (eNOS) [14]. Adiponectin—by activating eNOS and suppressing inducible NOS (iNOS) activity to limit hyperlipidemic vascular injury—also alleviates aortic stiffness, leading to endothelial function improvement [15]. The properties mentioned above could modulate vascular remodeling to prevent ED progression.

Circulating adiponectin concentrations may be used as a biomarker for kidney disease outcome evaluations [16]. Given that adiponectin levels might play a role in the ED that contributes to CV disease in CKD patients, the use of circulating adiponectin as an independent marker for ED in non-dialysis CKD populations remains uncertain. Therefore, the aim of our study is to investigate the relationship between the VRI, a validated non-invasive measure of endothelial function obtained through digital thermal monitoring (DTM) in humans, and adiponectin levels, as well as other traditional CV risk factors among stages 1–5 CKD patients. In addition, to confirm that adiponectin can ameliorate endothelial dysfunction in CKD mice from 5/6 nephrectomy as similar findings in human study. We further used an in vitro blood vessel myography technique to investigate whether adiponectin could enhance acetylcholine-induced vasodilation in CKD mice.

## 2. Materials and Methods

### 2.1. Participants

Between October 2017 and March 2019, we enrolled a total of 130 CKD participants in a medical center in Hualien, eastern Taiwan. The Research Ethics Committee, Hualien Tzu Chi Hospital, Buddhist Tzu Chi Medical Foundation (IRB106-108-A) approved the study. We acquired written informed consent from all participants before enrolling them in this study. The inclusion criteria included age > 18 years and presence of CKD for at least 3 months according to the Kidney Disease Outcomes Quality Initiative criteria. We measured blood pressure (BP) using standard mercury sphygmomanometers in the morning after the participants had sat for at least 10 min. We measured systolic BP (SBP) and diastolic BP (DBP) at the points of appearance and disappearance, respectively, three times at 5 min intervals, and averaged the results for analysis. We diagnosed hypertension (HTN) with an average BP ≥ 140/90 mmHg or if a patient had been taking antihypertensive medications in the previous two weeks. We defined diabetes mellitus (DM) as either a fasting plasma glucose of ≥126 mg/dL, a 2 h glucose level during an oral glucose tolerance test of ≥200 mg/dL, or use by a patient of oral hypoglycemic medications or insulin. We excluded patients with acute myocardial infarction, heart failure, chronic obstructive pulmonary disease, malignancy, or acute infection at the time of blood sampling, or if they refused to provide informed consent for the study.

### 2.2. Anthropometric Analysis

We measured the body weight of each participant in light clothing and without shoes to the nearest 0.5 kg, and assessed the body height to the nearest 0.5 cm. We measured waist circumference using a tape measure around the waist from between the lowest ribs and the hip bones with the hands on the hips. We calculated the body mass index as the weight in kilograms divided by the height in meters squared [9,17,18].

### 2.3. Biochemical Investigations and CKD Stage

We immediately centrifuged approximately 5 mL overnight fasting blood samples of the study participants at 3000× *g* for 10 min and then examined them with an auto-analyzer (Siemens Advia 1800, Siemens Healthcare GmbH, Henkestr, Germany) for the measurements of serum total cholesterol (TG), triglycerides (TCH), low-density lipoprotein cholesterol (LDL-C), fasting glucose, blood urea nitrogen (BUN), creatinine, uric acid, total calcium, and phosphorus levels [9,17,18]. We measured serum adiponectin concentrations using a commercially available enzyme immunoassay (SPI-BIO, Montigny le Bretonneux, France) [19]. We calculated estimated glomerular filtration rate (eGFR) using the mean value of measurements at least three months apart via the Chronic Kidney Disease Epidemiology Collaboration (CKD-EPI) equation. We divided the patients into different CKD stages according to the Kidney Disease Outcomes Quality Initiative criteria [9,18].

### 2.4. Endothelial Function Measurements

We obtained endothelial function tested by DTM via an FDA-approved device (VENDYS-II, Endothelix Inc., Houston, TX, USA) after an overnight fast and abstinence from caffeine, alcohol, tobacco, or vasoactive medications. After patients had rested at an ambient temperature of 22–24 °C for 30 min in a supine position, we placed BP cuffs and skin temperature sensors on the subject’s upper arms and the subject’s index fingers, respectively [18,20]. We performed DTM measurements of both hands during 5 min stabilization, 5 min cuff inflation to 50 mmHg > SBP, and 5 min deflation. Once the cuff was deflated, blood flow rushed into the forearm and hand, causing a temperature rebound in the fingertips directly proportional to the reactive hyperemia response. We determined VRI using VENDYS software II (VENDYS Corp., Houston, TX, USA) by taking the maximum difference between the observed temperature rebound curve and the zero-reactivity curve during the reactive hyperemia period. We classified patients as having good, intermediate, or poor VRI if VRI was ≥2.0, 1.0 to <2.0, or 0.0 to <1.0, respectively [9,19].

### 2.5. Animal Model of Chronic Kidney Disease by 5/6 Nephrectomy

Sixteen 8-week-old male C57BL6 mice weighing between 20 and 25 g were purchased from BioLASCO (Taipei, Taiwan) and approved by the Hualien Tzu Chi Hospital Animal Care and Use Committee. All mice were housed at the Tzu Chi University Laboratory Animal Centre (Hualien, Taiwan). The animals were kept in a temperature-controlled animal facility with a 12 h light/dark cycle, with water and food ad libitum. These mice were randomly assigned to C57BL/6 mice of sham operation (*n* = 8) and C57BL/6 mice of 5/6 nephrectomy (*n* = 8). All operation procedures were carried out using aseptic techniques. A 5/6 nephrectomy was performed in mice in two phases of surgery as previously described [20]. In summary, mice were anesthetized with isoflurane inhalation (Forane, Baxter, Deerfield, IL, USA) using a vaporizer (Matrx VIP 3000, Midmark, Versailles, OH, USA). Anesthesia was performed by a medium-line abdominal incision to expose the two kidneys, the right kidney was removed in the first phase, and the upper branch of the left kidney artery was bound 7 days later with a catgut absorbable suture. The abdominal muscle layer and skin were closed with a 5-0 sterile absorbent suture using a simple continuous technique. The sham group also underwent two-step surgery, but without the right kidney resection or binding of the upper branch of the left kidney artery. After surgery, mice were administered 2 mg/kg/day ketoprofen under their skin to alleviate pain. After 8 weeks, mice were sacrificed and thoracic aortic rings were immediately removed. These thoracic aortic segments were then submerged in Krebs’ solution [21,22]. After careful removal of fat and loose connective tissue, the thoracic aortic segments were cut into 4 mm long rings, which were subsequently mounted on stainless steel hooks and maintained at 37 °C while being aerated with a mixture of 95% O_2_ and 5% CO_2_. The thoracic aortic rings from the C57BL/6 mice of the sham operation were then divided into Krebs’ solution (control group, *n* = 4) or Krebs’ solution with adiponectin (10 μg/mL; ProSpec, East Brunswick, NJ, USA) (adiponectin group). The thoracic aortic rings of the C57BL/6 mice of the 5/6 nephrectomy were then divided into Krebs’ solution (CKD group, *n* = 4) or Krebs’ solution with adiponectin (CKD + adiponectin group).

### 2.6. Endothelial Function Measurements Assessment of Vascular Tension Reactivity Using In Vitro Blood-Vessel Myography

To evaluate the reactivity of vascular tension, an in vitro blood vessel myography technique was used. The procedures used in this case were using blood vessel myography systems as described above [21,22]. The tension changes in the thoracic aortic ring were measured using an isometric transducer (FT03C; West Warwick, RI, USA) and recorded using PowerLab’s data acquisition system (ADInstruments, Sydney, NSW, Australia). The optimal preload is established and the thoracic aortic ring stabilizes for about 60 min. After rinsing the Krebs solution, the segments were exposed to the adiponectin or the solvent for 15 min in the chamber. The function of endothelial cells was confirmed by assessing the relaxation response induced by acetylcholine (10^−9^ to 10^−5^ M, Sigma Aldrich, St. Louis, MO, USA) in the arteries pre-contracted with phenylephrine (10^−6^ M, Sigma Aldrich, St. Louis, MO, USA). A reference point is determined by generating a 100% contraction response to 75 mM KCl treatment and used to calculate the percentage of contraction changes for subsequent treatments [21,23].

### 2.7. Statistical Analysis

We tested the collected data for normal distribution using the Kolmogorov–Smirnov test. We expressed normally distributed data as the mean ± standard deviation and compared them using the Student’s independent *t*-test (two-tailed). We analyzed significant differences in measured values between groups using Kruskal–Wallis analysis or one-way analysis of variance followed by the Fisher’s protected *t*-test for parameters with and without normal distribution. Adiponectin levels among different CKD stages were further analyzed by the Cochran–Armitage test for trend. Because parameters including triglyceride, fasting glucose, BUN, and creatinine levels were presented with non-normal distribution, we logarithmically transformed these variables before further analysis. We evaluated variables correlated with VRI values by univariate linear regression analysis, and further analyzed variables with significant results in the univariate linear regression analysis, including Log-BUN, eGFR, and adiponectin with multivariate stepwise linear regression analysis. Spearman’s rank correlation coefficient was used to analyze the correlation between clinical variables and adiponectin levels. We performed analyses using SPSS for Windows (version 19.0; SPSS Inc., Chicago, IL, USA). We defined a *p* value < 0.05 as statistically significant.

## 3. Results

In Table 1, we present the clinical characteristics for patients with CKD. Among 130 patients with CKD, 55 (42.3%) were female, 65 (50.0%) had DM, and 105 (80.8%) had HTN. In these CKD populations, we diagnosed 22 (16.9%), 39 (30.0%), and 69 (53.1%) individually with poor, intermediate, and good VRI, respectively. As the VRI decreased among CKD subjects, the patient’s adiponectin (*p* < 0.001) and eGFR (*p* = 0.047) levels decreased significantly, whereas their serum BUN (*p* = 0.037) level increased significantly. Sex, DM, and HTN were not linked to significant differences among the three groups of patients.

By univariate linear regression analysis, we detected a negative correlation between logarithmically transformed BUN (log-BUN, *r* = −0.244, *p* = 0.005), and a positive correlation between serum adiponectin (*r* = 0.512, *p* < 0.001) and eGFR (*r* = 0.230, *p* = 0.008), respectively, and VRI values in patients with CKD (Table 2). In addition, adjustments for variables significantly associated with VRI values in univariate linear regression analysis revealed a significant and independent negative, and a significant independent positive association between log-BUN (standardized β = −0.176, adjusted R^2^ change = 0.025; *p* = 0.021), and adiponectin (standardized β = 0.487, adjusted R^2^ change = 0.256; *p* < 0.001), respectively, and VRI values in patients with CKD by multivariate stepwise linear regression analysis (Table 2).

**Figure 1:**
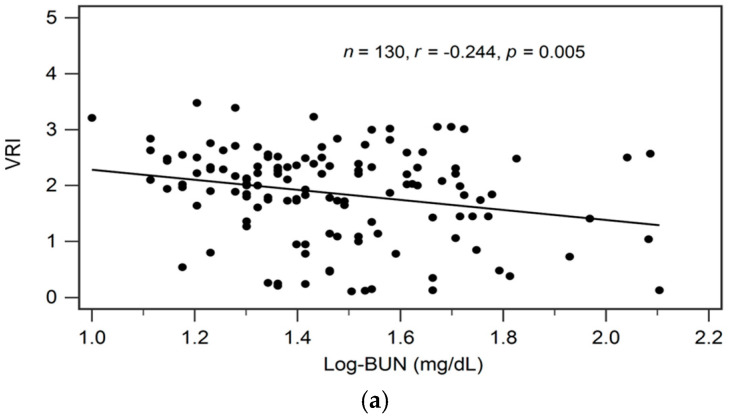

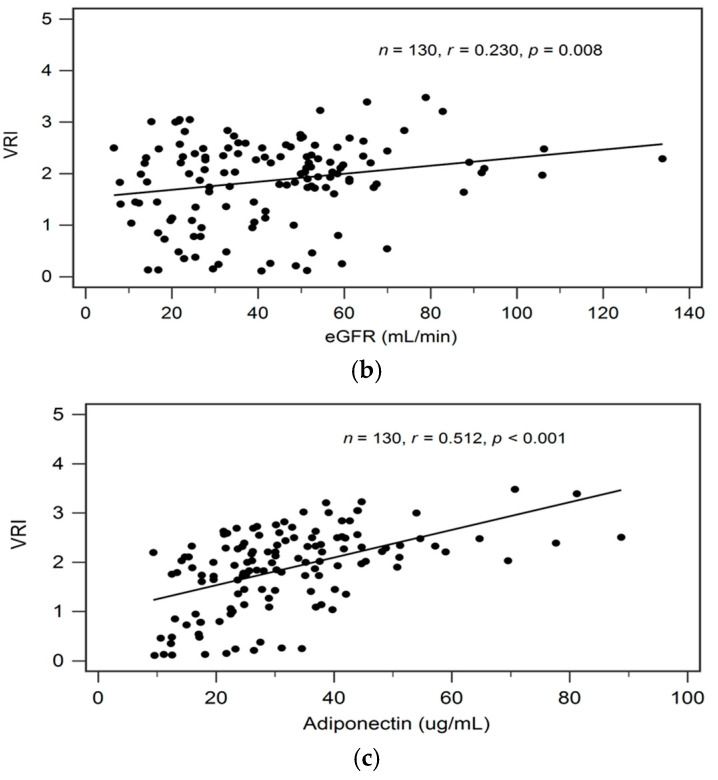
Relationships between vascular reactive index (VRI) and (**a**) log-BUN level (ng/mL), (**b**) eGFR (mL/min), or (**c**) adiponectin level (ng/mL) among 130 chronic kidney disease patients.

In Figure 1a–c we present two-dimensional scattered plots of VRI values with serum log-BUN, eGFR, and serum adiponectin levels, respectively, among CKD stages 1–5 patients.

Table 3 shows the correlation between clinical variables and serum adiponectin level by Spearman’s correlation analysis. In addition, known serum adiponectin level is positively associated with VRI (*r* = 0.512, *p* < 0.001); serum adiponectin levels also showed significantly negative correlation with BMI (*r* = −0.197, *p* = 0.025), waist circumference (*r* = −0.285, *p* = 0.001), body fat mass (*r* = −0.185, *p* = 0.035), log-TG (*r* = −0.182, *p* = 0.038), log-glucose (*r* = −0.183, *p* = 0.037), and eGFR (*r* = −0.277, *p* = 0.001).

**Figure 2:**
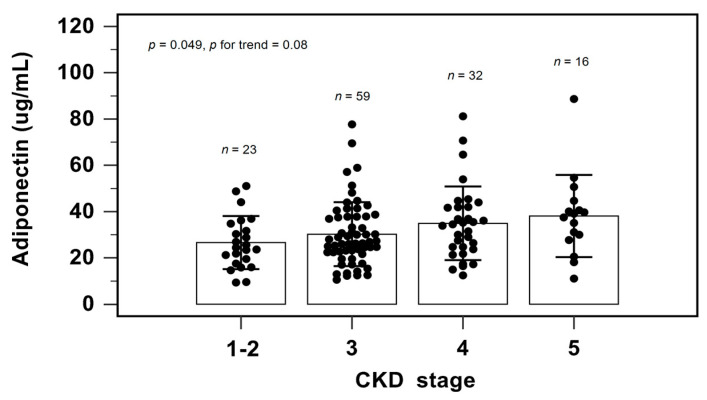
Serum levels of adiponectin to CKD staging.

Figure 2 shows the serum adiponectin level among different CKD stages by one-way analysis of variance and the Cochran–Armitage test for trend. As the CKD stage progressed, the serum adiponectin levels elevated (*p* = 0.049). The Cochran–Armitage test for trend also confirmed this finding (*p* = 0.008).

**Figure 3:**
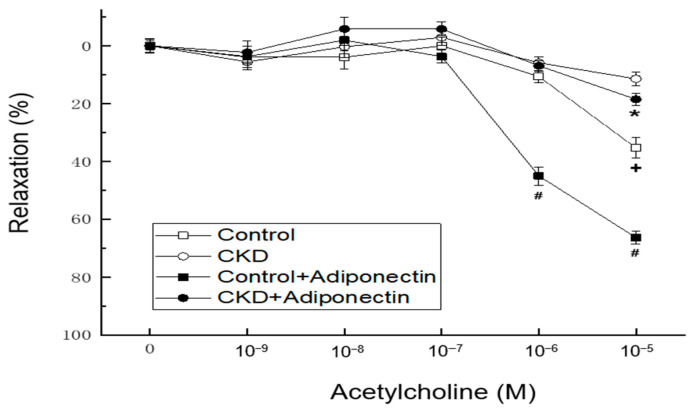
Adiponectin treatment ameliorates the CKD-induced hyporeactivity to vasorelaxation in the mouse aorta. * *p* < 0.05 for the CKD + adiponectin group compared with the CKD group. # *p* < 0.05 for the adiponectin group compared with control group. + *p* < 0.05 for the CKD group compared with the control group.

In vascular tension reactivity using in vitro blood-vessel myography, CKD mice had poorer vasorelaxation induced by acetylcholine at concentrations of 10^−5^ M than the control group (Figure 3, + *p* < 0.05). Compared to the control group, the administration of adiponectin enhanced vasorelaxation in response to acetylcholine at concentrations of 10^−6^ and 10^−5^ M at adiponectin group (Figure 2, # *p* < 0.05). Moreover, in the thoracic aortic segments of mice with CKD, adiponectin further augmented the vasorelaxation induced by acetylcholine at a concentration of 10^−5^ M at CKD + adiponectin group (CKD + adiponectin group: 18.6 ± 2.0% vs. CKD group: 11.5 ± 2.5%, * *p* < 0.05, as shown in Figure 2). These findings collectively provide evidence supporting the role of adiponectin in enhancing acetylcholine-mediated vasorelaxation, the impaired vasorelaxation associated with CKD, and the beneficial effect of adiponectin in ameliorating the reduced vasorelaxation caused by CKD.

## 4. Discussion

This study showed that BUN had a negative, and eGFR and adiponectin levels had a positive association with VRI values assessed by DTM among CKD patients. Moreover, the associations between both serum adiponectin and log-BUN levels, and VRI levels remained significant and independent after adjusting for confounders. In an animal study also noted adiponectin improved the vasorelaxation induced by acetylcholine in CKD mice of 5/6 nephrectomy.

ED, a chief initial event in the atherosclerosis pathogenesis presented as endothelium-dependent vasodilation impairment, decreased nitric oxide (NO) bioavailability and increased pro-thrombotic properties [24,25], and had a strong correlation with the presence of many CV diseases, including coronary artery disease, chronic heart failure, and CKD [6]. Evaluating endothelium function via non-invasive methods or biomarkers has attracted strong interest in the assessment of adverse outcomes in CKD subjects [26]. Recently, the VRI derived from DTM has provided an easier non-operator-dependent and non-invasive method for assessing the microvascular function of patients [19].

Adiponectin functions as an insulin sensitizer, overcoming the insulin resistance that plays a role in the pathogenesis of ED. Insulin is suggested to stimulate endothelial NO production and serve as a vasodilator [27]. Insulin also regulates vascular function by stimulating the expression of vascular cell adhesion molecules, soluble intercellular cell adhesion molecule-1 (iCAM-1), soluble vascular cell adhesion molecule-1 (vCAM-1), and E-selectin on the endothelium [28]. Serum adiponectin concentration was previously reported to be positively associated with soluble vCAM-1 level and was associated with the ED measured by the flow-mediated dilatation in type 2 DM patients with diabetic nephropathy [29]. Another possible association between the ED and insulin resistance may further be associated due to the suppressed dihydropterin reductase activity caused by insulin resistance with the subsequent depletion of tetrahydrobiopterin (BH4)—a key cofactor of the catalytic activity of NOS [30]. Decreases in BH4 levels might elevate oxidative stress and cause ED [6]. Taken together, hypoadiponectinemia is a significant factor in insulin resistance, a risk factor for ED occurrence and progression. Our study showed that serum adiponectin levels were positively associated with VRI values after adjustment for confounders in patients with CKD.

The uremic milieu can disrupt adipocyte metabolism, leading to pathogenic consequences. In CKD populations, increased adiponectin production occurs in both visceral and subcutaneous adipose tissues, while circulating adiponectin levels in pre-dialysis CKD patients negatively correlate with eGFR [31]. Nevertheless, the impact of elevated adiponectin levels on CKD patients’ metabolism remains uncertain. One plausible hypothesis for the association between high serum adiponectin concentrations and adverse CV outcomes in CKD patients is the development of adiponectin resistance, especially among those undergoing dialysis, owing to the uremic environment. An alternative explanation is that adiponectin secretion rises significantly to counterbalance the effects of heightened inflammatory cytokines and/or vascular injuries, but the inflammatory environments and proatherogenic uremic might overpower its effects [31]. Our study showed that as the CKD stage progressed, the serum adiponectin levels elevated and serum adiponectin levels are negatively associated with eGFR. Adiponectin has insulin-sensitizing, anti-diabetic, and anti-atherogenic effects [11]. Our study also showed serum adiponectin levels are negatively associated with BMI, waist circumference, body fat mass, log-TG, and log-glucose in this study.

The effects of hypoadiponectinemia on the pathogenesis of the ED are likely to be multifactorial. NO, which reduces vasoconstriction and blocks endothelial apoptosis and plaque formation, is an important signaling molecule involved in the vasculo-protective role of adiponectin [32]. On endothelial cells, adiponectin can induce an adenosine monophosphate-activated protein kinase (AMPK)- and cAMP-protein kinase A (PKA)-signaling pathway to regulate vascular homeostasis. Adiponectin can activate AMPK-eNOS, which increases NO production and causes relaxation of the endothelium [33]. By triggering AMPK-eNOS, adiponectin can also promote the endothelial progenitor cells to differentiate into endothelial cells, which plays a pivotal role in vascular protection [34]. Additionally, adiponectin exhibits anti-inflammatory effects via activating cAMP-PKA signaling, which in turn both suppresses ROS generation and NF-κB signaling and promotes NO production in the vascular endothelium [33]. Moreover, adiponectin improves endothelial function via its anti-atherosclerotic mechanism by the suppression of iNOS and the activation of eNOS activity in the vasculature to alleviate hyperlipidemic vessel injury-related aortic stiffness [15]. On human macrophages, adiponectin both interrupts the activation of the M1 macrophage, which has proinflammatory effects, and promotes cellular differentiation of monocytes to M2 macrophages, which can block an inflammatory response [35]. Further, adiponectin elevates anti-inflammatory factor IL-10 levels and suppresses proinflammatory cytokines such as IL-6, TNF-α, and IFN-γ in macrophages [36]. The modulating vascular remodeling properties of adiponectin mentioned above can prevent the progression of ED. Our study also revealed a significant association between hypoadiponectinemia and ED among non-dialysis CKD patients and in animal study also noted adiponectin enhancing acetylcholine-mediated vasorelaxation using in vitro blood-vessel myography in 5/6 nephrectomy CKD mice.

Our study has some limitations. First, the study was cross-sectional, with a limited number of CKD patients at a single hospital; it therefore failed to elucidate the causal relationship between serum adiponectin values and the presence of ED in the non-dialysis CKD population. Long-term large sample size prospective studies to investigate the causal relationship of our findings are necessary. Second, this study measured serum total adiponectin concentrations and recent studies have revealed that different adiponectin isoforms can represent both pro-inflammatory and anti-inflammatory functions, and the ratio between high-molecular-weight (HMW) and low-molecular-weight adiponectin is an essential biomarker of CVD occurrence [11,37]. However, concerns remain about the absence of a universal standard and the inability to accurately measure adiponectin isoforms [11]. Although previous studies had demonstrated that HMW adiponectin is the most potent isoform, a strong association between serum HMW and total adiponectin was reported [38]. Moreover, both serum HMW and total adiponectin concentrations have been inversely associated with parameters of ED, insulin resistance, and inflammation, indicating that the use of total adiponectin level is a well-accepted surrogate of endothelial function evaluation [38]. Third, previous data have revealed that age, sex, BP, albuminuria, and smoking are essential determinants of ED [6,39]. We did not evaluate some confounders, such as albuminuria and smoking status, known to affect the ED occurrence, in our study, which may have restricted its predictive power. The other parameters of age, gender, and BP were not associated with vascular reactivity measured by DTM among CKD subjects in our study, probably due to the high comorbidity of the studied cohort, making it difficult to separate the contributions of these factors from other contributing mechanisms [2,40].

## 5. Conclusions

Here, we revealed that serum adiponectin levels—an emerging potential biomarker for ED—were an independent positive predictor of ED in non-dialysis CKD patients. Adiponectin also helps improve reduced vasorelaxation caused by acetylcholine in 5/6 nephrectomy CKD mice. These findings support adiponectin’s role in enhancing vasorelaxation, particularly in individuals with CKD. Further prospective studies are indicated to confirm the mechanisms underlying this association.

## Figures and Tables

**Table 1:** Clinical characteristics according to different vascular reactivity index by digital thermal monitoring of the 130 chronic kidney disease patients.

Characteristics	All Patients (*n* = 130)	Good Vascular Reactivity (*n* = 69)	Intermediate Vascular Reactivity (*n* = 39)	Poor Vascular Reactivity (*n* = 22)	*p* Value
Age (years)	65.35 ± 11.94	63.65 ± 10.36	67.87 ± 13.40	66.23 ± 13.42	0.197
Height (cm)	159.69 ± 8.49	160.61 ± 8.11	158.59 ± 9.45	158.75 ± 7.91	0.423
Body weight (kg)	68.70 ± 14.55	69.12 ± 14.31	70.05 ± 16.31	64.99 ± 11.71	0.405
Body mass index (kg/m^2^)	26.79 ± 4.38	26.65 ± 4.39	27.65 ± 4.61	25.72 ± 3.80	0.239
Waist circumference (cm)	88.44 ± 11.27	87.87 ± 11.83	90.05 ± 10.91	87.36 ± 10.27	0.456
Body fat mass (%)	30.94 ± 7.60	30.81 ± 7.69	31.41 ± 7.40	30.50 ± 7.99	0.887
VRI	2.02 (1.40–2.45)	2.39 (2.21–2.66)	1.73 (1.36–1.83)	0.42 (0.18–0.78)	<0.001 *
SBP (mmHg)	135.92 ± 16.27	135.86 ± 14.81	135.49 ± 16.28	136.91 ± 20.84	0.947
DBP (mmHg)	76.91 ± 11.28	78.49 ± 10.08	73.87 ± 11.25	77.32 ± 14.07	0.121
TCH (mg/dL)	157.14 ± 34.95	155.32 ± 33.21	156.74 ± 37.47	163.55 ± 36.62	0.631
TG (mg/dL)	121.50 (94.00–172.50)	117.00 (95.00–170.50)	122.00 (86.00–162.00)	125.00 (93.25–214.50)	0.646
LDL-C (mg/dL)	90.21 ± 31.23	90.23 ± 30.49	88.38 ± 32.51	93.36 ± 32.42	0.838
Fasting glucose (mg/dL)	101.50 (98.00–130.50)	106.00 (100.00–130.50)	100.00 (98.00–150.00)	100.50 (94.75–150.75)	0.195
BUN (mg/dL)	27.00 (20.00–41.00)	24.00 (19.00–38.00)	30.00 (21.00–51.00)	30.50 (24.50–48.50)	0.037 *
Creatinine (mg/dL)	1.60 (1.20–2.4)	1.50 (1.20–2.35)	1.60 (1.20–2.40)	2.20 (1.38–2.70)	0.129
eGFR (mL/min)	41.51 ± 21.98	45.65 ± 22.99	38.56 ± 21.69	33.55 ± 16.01	0.047 *
Uric acid (mg/dL)	5.97 ± 1.73	5.80 ± 1.57	6.25 ± 2.04	5.97 ± 1.62	0.437
Total calcium (mg/dL)	9.58 ± 1.52	9.70 ± 1.51	9.21 ± 1.32	9.87 ± 1.81	0.175
Phosphorus (mg/dL)	3.86 ± 0.66	3.88 ± 0.68	3.89 ± 0.59	3.73 ± 0.73	0.592
Adiponectin (μg/mL)	31.74 ± 14.81	37.65 ± 16.12	28.73 ± 8.93	18.53 ± 6.81	<0.001 *
Female, *n* (%)	55 (42.3)	29 (42.0)	17 (43.6)	9 (40.9)	0.977
DM, *n* (%)	65 (50.0)	38 (55.1)	18 (46.2)	9 (40.9)	0.434
HTN, *n* (%)	105 (80.8)	53 (76.8)	34 (87.2)	18 (81.8)	0.418
CKD stage 1, *n* (%)	5 (3.9)	4 (5.8)	1 (2.6)	0	0.263
CKD stage 2, *n* (%)	18 (13.8)	13 (18.8)	4 (10.3)	1 (4.5)	
CKD stage 3, *n* (%)	60 (46.2)	32 (46.4)	18 (46.2)	10 (45.5)	
CKD stage 4, *n* (%)	31 (23.8)	15 (21.7)	8 (20.5)	8 (36.4)	
CKD stage 5, *n* (%)	16 (12.3)	5 (7.2)	8 (20.5)	3 (13.6)	

Values for continuous variables given as means ± standard deviation and test by one-way analysis of variance; variables not normally distributed given as medians and interquartile range and test by Kruskal–Wallis analysis; values are presented as number (%) and analysis after analysis by chi-square test. SBP, systolic blood pressure; DBP, diastolic blood pressure; TCH, total cholesterol; TG, triglyceride; LDL-C, low-density lipoprotein cholesterol; eGFR, estimated glomerular filtration rate; CKD, chronic kidney disease; VRI, vascular reactivity index; BUN, blood urea nitrogen; DM, diabetes mellitus; HTN, hypertension. * *p* < 0.05 was considered statistically significant.

**Table 2:** Correlation of vascular reactivity index levels and clinical variables by simple or multivariable linear analyses among 130 chronic kidney disease patients.

Variables	Vascular Reactivity Index				
	Simple Linear Regression		Multivariable Linear Regression		
	*r*	*p* Value	Beta	Adjusted R^2^ Change	*p* Value
Female	0.040	0.655	–	–	–
DM	0.066	0.454	–	–	–
HTN	−0.031	0.723	–	–	–
Age (years)	−0.121	0.171	–	–	–
Height (cm)	0.038	0.677	–	–	–
Body weight (kg)	0.031	0.728	–	–	–
Body mass index (kg/m^2^)	0.019	0.829	–	–	–
Waist circumference (cm)	−0.087	0.327	–	–	–
Body fat mass (%)	0.032	0.718	–	–	–
Systolic blood pressure (mmHg)	−0.028	0.754	–	–	–
Diastolic blood pressure (mmHg)	0.092	0.300	–	–	–
TCH (mg/dL)	0.010	0.914	–	–	–
Log-TG (mg/dL)	-0.012	0.891	–	–	–
LDL-C (mg/dL)	0.051	0.563	–	–	–
Log-Glucose (mg/dL)	0.056	0.527	–	–	–
Log-BUN (mg/dL)	−0.244	0.005 *	−0.176	0.025	0.021 *
Log-Creatinine (mg/dL)	−0.200	0.023 *	–	–	–
eGFR (mL/min)	0.230	0.008 *	–	–	–
Uric acid (mg/dL)	−0.034	0.699	–	–	–
Total calcium (mg/dL)	0.043	0.631	–	–	–
Phosphorus (mg/dL)	0.050	0.574	–	–	–
Adiponectin (μg/mL)	0.512	<0.001 *	0.487	0.256	<0.001 *

Data of TG, fasting glucose, BUN, and creatinine showed skewed distribution and therefore were log-transformed before analysis. Analysis of data was performed using the simple linear regression analyses or multivariable stepwise linear regression analysis (adapted factors were log-BUN, log-Creatinine, eGFR and adiponectin). DM, diabetes mellitus; HTN, hypertension; TCH, total cholesterol; TG, triglyceride; LDL-C, low-density lipoprotein cholesterol; BUN, blood urea nitrogen; eGFR, estimated glomerular filtration rate. * *p* < 0.05 was considered statistically significant.

**Table 3:** Correction between adiponectin level and clinical variables.

Variables	Spearman’s Correlation Coefficient	*p* Value
VRI	0.512	<0.001 *
Age (years)	−0.078	0.379
BMI (kg/m^2^)	−0.197	0.025 *
Waist circumference (cm)	−0.285	0.001 *
Body fat mass (%)	−0.185	0.035 *
TCH (mg/dL)	0.044	0.618
Log-TG (mg/dL)	−0.182	0.038 *
LDL-C (mg/dL)	0.005	0.959
Log-Glucose (mg/dL)	−0.183	0.037 *
eGFR (mL/min)	−0.277	0.001 *
Uric acid (mg/dL)	−0.026	0.772
Total calcium (mg/dL)	−0.008	0.931
Phosphorus (mg/dL)	0.084	0.343
SBP (mmHg)	0.053	0.548
DBP (mmHg)	0.132	0.133

Data of glucose and triglyceride levels showed skewed distribution, and therefore were log-transformed before analysis. Analysis of data was performed using the Spearman correlation analysis. VRI, vascular reactive index; BMI, body mass index; eGFR, estimated glomerular filtration rate; TCH, total cholesterol; TG, triglyceride; LDL-C, low-density lipoprotein cholesterol; SBP, systolic blood pressure; DBP, diastolic blood pressure. * *p* < 0.05 was considered statistically significant (2-tailed).

## Data Availability

The data that support the findings of this study are available on request from the corresponding author.
